# The Fumigation of an Ambassador

**Published:** 1897-05-08

**Authors:** 


### The Hospital

A Journal of

The Medical Sciences and Hospital Administration.

Vol. XXII.—No. 554.] [May 8, 1897.

### THE FUMIGATION OF AN AMBASSADOR.

Thackeray somewhere says that "we preach, and every man furnishes the example for his own sermon." It would be difficult to find a better illustration of the great humorist's dictum than that which has just been furnished by the sanitary authorities who are clothed with a little, and, we trust, also with a "brief" authority, in the province of British Columbia. These doubtless worthy persons, at the very time when the chief sanitary authority of Great Britain, Dr. Thorne-Thorne, C.B., has recently returned from an International Conference at Venice, at which he succeeded in persuading the representatives of sixteen powers to accept British methods of dealing with imported contagions, have thought fit to arrest the progress of His Excellency Chang Hin Yuan, the special Chinese Envoy, to the celebration of the Queen's sixty years of reign, and have even proposed to submit His Excellency himself to a process of "fumigation," on no other or better ground than the circumstance that the vessel in which His Excellency and his suite made the voyage from China has had a case of small-pox on board. The scanty information which has so far reached this country by telegraph does not allow us to say whether the subject of the disease was a member of the ambassadorial suite, a sailor, or a passenger; or at what period of the voyage the malady declared itself. None of these facts, however, are in the least degree material to the formation of a judgment on the main issue concerned, nor could they, whatever they might be, either diminish or increase the absurdity of the action which is briefly chronicled. We ourselves have abandoned quarantine as absolutely useless; the final remnants of our old quarantine laws were abolished in the last session of Parliament; and yet our kinsmen and fellow subjects on the western boundary of the Dominion of Canada are proposing to put this antiquated restriction in force against a personage invested by his high office with peculiar privileges, and, therefore, in circumstances which confer upon the attempt an importance calculated to raise it above the level of being merely ridiculous.

We have no information as to the manner in which the mysterious process of "fumigation" is conducted in the remote region in which some faith in it appears still to linger, nor do we know whether its effectual performance would render necessary the undoing of the sacred strands of the ambassadorial pigtail, so that the material employed, whatever it might be, should be enabled to penetrate to even the innermost hairs of this conceivable *fomes*. Nor is our curiosity likely to be satisfied by any description of the actual operation, since His Excellency is understood to object to it, not at all as a matter of common sense, but purely as a matter of dignity, and to assert that he will return to China, and suffer decapitation as the natural consequence of his failure to discharge the duties of the high office committed to him by his sovereign, rather than allow the profane hands of sanitary officials to do violence to his person. In this resolution he has our entire sympathy.

By way of contrast to this silly business, and as an immediate consequence of the Vienna Conference, it may be mentioned that a transport from India, carrying 1,200 men, women, and children, reached Southampton a month ago, having had on board a case of plague which terminated fatally before she arrived at Suez. On her arrival at Southampton, all on board were examined, and found healthy; and, following English practice, none were detained; for, just as we object to consider any man guilty because he has not absolutely proved his innocence, so we object to consider that he is in the incubation stage of an infectious disease merely because it is not impossible that he may be so. The parts of the vessel which the plague patient had occupied were disinfected, and the incident was at an end. The vessel in question was the third which had come to this country plague infected, and from plague-infected ports in India; and in none of the instances has there been any appearance of the disease upon our shores. It was in consequence of the resolutions of the Venice Conference that these ships were allowed to pass through the Suez Canal in quarantine, that is, with guards on board, and to complete the period during their passage up the Mediterranean. It is high time that the Dominion Government should bring itself within the "Concert of Europe," and should realise, as we in England have done long ago, that quarantine is totally condemned by abundant experience, that it is useless against disease, costly and harassing to travellers, and disastrous in its effects upon the shippers of goods; while a belief in the fumigation of persons, not to speak of ambassadors, can only be regarded as a survival from the dark ages, analogous to a belief in sorcery or witchcraft. We are infinitely proud of our great colonial empire, and we are especially proud of the Dominion which has just relaxed her restrictions upon imports in our favour; but we are all the more solicitous that she should guide her footsteps by the light of science and experience, and should refrain from rendering herself ridiculous even in the cause of sanitation.

